# 100 DEFRAIL: The Development of a Patient-centred, Theory-informed, Expert-guided Diet and Exercise for Frailty Intervention

**DOI:** 10.1093/eurpub/ckae114.055

**Published:** 2024-09-26

**Authors:** Sarah Fagan, John Cooke, Ríona Mulcahy, Padraig Bambrick, Michael Harrison, Brian Mulhare, Bróna Kehoe

**Affiliations:** South East Technological University, Waterford, Ireland; Waterford Integrated Care for Older People (WICOP), Department of Geriatric Medicine, University Hospital Waterford, Ireland; Department of Medicine, Royal College of Surgeons in Ireland, Ireland; Waterford Integrated Care for Older People (WICOP), Department of Geriatric Medicine, University Hospital Waterford, Ireland; Department of Medicine, Royal College of Surgeons in Ireland, Ireland; Waterford Integrated Care for Older People (WICOP), Department of Geriatric Medicine, University Hospital Waterford, Ireland; Department of Medicine, Royal College of Surgeons in Ireland, Ireland; South East Technological University, Waterford, Ireland; South East Technological University, Waterford, Ireland; South East Technological University, Waterford, Ireland

## Abstract

**Purpose:**

Combined exercise and dietary interventions have been shown to benefit older adults with frailty, with significant promise in transitioning between frailty states. However, there is limited availability of such interventions, partly due to a lack of capacity in healthcare. While community-based programmes offer a plausible solution, often intervention settings do not reflect the transfer of research to practical ‘real world’ programmes and there is a paucity of research investigating ‘true’ community-based programmes. With older adults citing numerous barriers to adherence, the purpose of this study was to describe the development of DEFRAIL (Diet and Exercise for Frailty), a patient-centred, evidenced-based behaviour change intervention.

**Methods:**

The research is guided by the Medical Research Council’s Framework for the Development, Evaluation, and Implementation of Complex Interventions. Four key steps contributed to the intervention development. 1) A review of the literature identified the evidence for intervention delivery models. 2) Interviews with older adults with frailty (n = 13) explored attitudes and preferences for exercise and protein supplementation. 3) A behaviour change analysis was performed using the COM-B Model to identify behaviour change techniques (BCTs) to embed in the intervention. 4) An expert panel of healthcare professionals, academics and potential implementers (n = 18) was consulted, via a DELPHI process to obtain consensus on the final structure of the intervention.

**Results:**

There is a lack of detail on intervention delivery models in the literature. Settings varied for ‘community’ interventions from universities, research centres, gym and home-based programmes. Three key themes were identified from the interviews with older adults: impact of physical limitations, impact of knowledge, impact of medical professionals, which influenced capability, opportunity, and motivation in regards to the behaviours. Modifiable barriers identified, informed the selection of intervention functions and BCTs, which were translated into actionable intervention components. The expert panel agreed the final components of the intervention.

**Conclusion:**

Developing interventions underpinned by evidence, theory and with input from end-users and experts will enhance the potential for positive behaviour change, promoting adherence to the intervention and hence, increase its potential effectiveness.

**Support/Funding Source:**

This study is funded by the Irish Research Council enterprise partner scholarship with industry partner Tírlan.

